# Expression profiling of primary and metastatic oral squamous cell carcinoma identifies progression‐associated transcriptome changes and therapeutic vulnerabilities

**DOI:** 10.1002/cac2.12660

**Published:** 2025-01-07

**Authors:** Jonas Pyko, Markus Glaß, Julia Rosemann, Matthias Kappler, Jana Macho, Sarah Qasem, Stefan Hüttelmaier, Alexander W. Eckert, Monika Haemmerle, Tony Gutschner

**Affiliations:** ^1^ Institute of Molecular Medicine, Section for RNA biology and pathogenesis Faculty of Medicine, Martin Luther University Halle‐Wittenberg Halle (Saale) Germany; ^2^ Institute of Molecular Medicine, Section for Molecular Cell Biology Faculty of Medicine Martin Luther University Halle‐Wittenberg Halle (Saale) Germany; ^3^ Department of Oral and Maxillofacial Plastic Surgery Faculty of Medicine, Martin Luther University Halle‐Wittenberg Halle (Saale) Germany; ^4^ Department of Cranio Maxillofacial Surgery Paracelsus Medical University Nuremberg Germany; ^5^ Institute of Pathology Section for Experimental Pathology Faculty of Medicine Martin Luther University Halle‐Wittenberg Halle (Saale) Germany; ^6^ Abbvie Deutschland GmbH & Co. KG Ludwigshafen am Rhein Germany

**List of abbreviation**
C1/2/3: cluster 1/2/3
CI: confidence interval
Ctrl: control
DFS: disease‐free survival
dIF: differences in isoform fraction
E2F: Early region 2 binding factor
EMT: Epithelial‐to‐Mesenchymal Transition
FDR: False Discovery Rate
GABRG3: Gamma‐Aminobutyric Acid Type A Receptor Subunit Gamma 3
GO: Gene Ontology
HNSCC: Head and Neck Squamous Cell Carcinoma
HR: Hazard Ratio
HTR6: 5‐Hydroxytryptamine Receptor 6
KRAS: Kirsten Rat Sarcoma Viral Oncogene Homolog
lncRNA: long non‐coding RNA
LNM: Lymph node metastasis / metastases
MAOB: Monoamine Oxidase B
MYC: Myelocytomatosis oncogene
MYL6: Myosin Light Chain 6
NES: Normalized Enrichment Score
OS: overall survival
OSCC: Oral Squamous Cell Carcinoma
RFS: recurrence‐free survival
RORC: RAR Related Orphan Receptor C
RPL7: Ribosomal Protein L7
RR: Relative Risk
SHMT2: Serine Hydroxymethyltransferase 2
siRNA: small interfering RNA
STAC3: SH3 And Cysteine Rich Domain 3
TCGA: The Cancer Genome Atlas
TUBB4A: Tubulin Beta 4A Class IVa
WNT5A: Wnt family member 5A
ZNF443: Zinc Finger Protein 443

1

Oral squamous cell carcinoma (OSCC), a major subgroup of head and neck squamous cell carcinoma (HNSCC), is an aggressive disease that preferentially spreads to cervical lymph nodes. Positive lymph node status is an important predictor of survival in OSCC [1, 2, 3]. Hence, a better understanding of the molecular mechanisms underlying oral cancer metastasis and the identification of therapeutic vulnerabilities are needed to prevent and treat metastatic disease.

We collected 87 primary tumors and 21 lymph node metastasis (LNM) from 72 OSCC patients to conduct comprehensive transcriptome‐wide expression and correlation analyses (Figure 1A). First, we performed expression‐based clustering with all primary tumors and observed the best subdivision with *k* = 3 using protein‐coding and non‐coding genes (Figure 1B). Of note, we observed transcriptional heterogeneity among multiregional tumor samples in about 30% of the cases, leading to the assignment of these patients and their respective tumors to different clusters. Intriguingly, Kaplan‐Meier analysis of patients whose tumors were unambiguously assigned to only one cluster revealed that cluster 3 (C3) had the worst outcome, with a median survival of 15.6 months compared to 28.57 and 36.53 months for clusters 1 (C1) and 2 (C2), respectively (Figure 1C). Importantly, prognostic factors known to negatively affect survival, such as high T, N, and G status, were not enriched in C3 tumors (Supplementary Figure S1A‐C). However, gene expression analysis identified 244 genes that were significantly changed in C3 compared to C1/2 tumors (Supplementary Figure S1D, Supplementary Table S1). Of note, cell cycle‐related gene sets, including Early region 2 binding factor (*E2F*) and Myelocytomatosis oncogene (*MYC*) target genes, along with other oncogenic signaling pathways, showed a positive normalized enrichment score (NES), potentially explaining the poor outcomes of C3 tumors (Supplementary Figure S1E).

**FIGURE 1:**
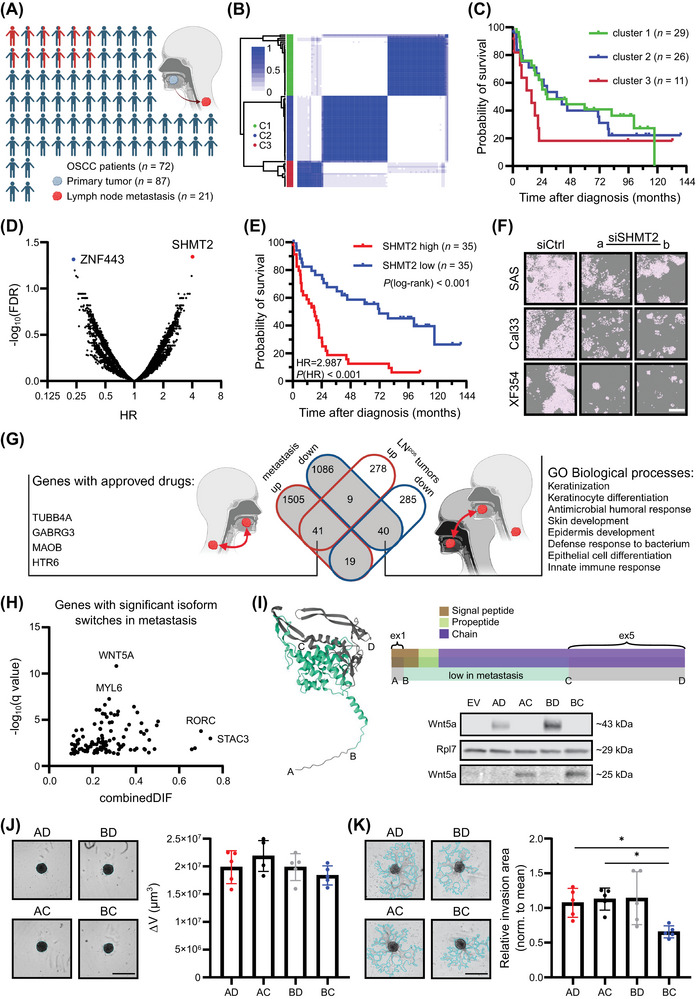
Deep transcriptome and isoform analysis reveals survival and metastasis biomarkers and targeting options for oral squamous cell carcinoma. (A) Overview of the Oral Squamous Cell Carcinoma (OSCC) patient and tissue cohort. The scheme was created in BioRender. Gutschner, T. (2025) https://BioRender.com/q66a024. (B) Consensus matrix of the primary tumor samples obtained via consensus clustering. Consensus values indicate the frequency with which samples clustered together (0 ‐ never to 1 ‐ always) during distinct permutations of the algorithm. (C) Kaplan‐Meier overall survival (OS) analysis of patients with tumors unambiguously assigned to one of three clusters. Patients with cluster 3 tumors tend to have worse OS. (D) Hazard ratio (HR) values plotted against False Discovery Rate (FDR)‐adjusted log‐rank test‐derived *P*‐values of all protein‐coding and non‐coding genes. Samples were divided by tertile separation, and the gene list was filtered for at least 10 samples per group (genes with FDR ≤ 0.05 are highlighted). (E) Kaplan‐Meier analysis of OS in the OSCC cohort (*n* = 70 patients) stratified by *SHMT2* mRNA abundance. Samples were divided by median separation. (F) Representative images of OSCC cell lines after transfection with two independent *SHMT2*‐targeting small interfering RNAs (siSHMT2a/b). Two‐dimensional growth was strongly impaired after depletion of *SHMT2* in all three cell lines. The white scale bar indicates 400 µm. (G) Venn diagram of significantly deregulated genes in metastases and LNM^pos^‐ tumors. Genes upregulated in both datasets and targeted by an approved drug, as well as gene ontology (GO) biological processes significantly overrepresented in downregulated genes found in both datasets, are indicated. The scheme was created in BioRender. Gutschner, T. (2025) https://BioRender.com/k79r687. (H) Plot of genes with significant isoform switches (q ≤ 0.05) in metastases, as obtained by the R package IsoformAnalyzeR. Genes with the highest combined differences in isoform fraction values (DIFs) and lowest q‐values are highlighted. (I) Three‐dimensional protein structure of the canonical WNT5A protein according to AlphaFold. The turquoise area marks the WNT5A‐203 isoform, which is decreased in lymph node metastases. Letter codes indicate primer binding sites for the cloned overexpression constructs used for functional assays. Representative Western blot (*n* = 3) shows the overexpression of different WNT5A protein isoforms in SAS cells. Ribosomal Protein L7 (RPL7) was used as a loading control. (J‐K) Representative pictures depicting spheroid growth (J) and matrigel‐based invasion (K) upon overexpression of the respective *WNT5A* isoforms in SAS cells (scale bars = 800 µm). Bar graphs show the mean and standard deviation (*n* = 5). One‐way ANOVA (Holm‐Sidak corrected) was performed for statistical testing, with *P ≤ 0.05. Abbreviations: C1/2/3, cluster 1/2/3; DIF, differences in isoform fraction; GABRG3, Gamma‐Aminobutyric Acid Type A Receptor Subunit Gamma 3; GO, gene ontology; FDR, False Discovery Rate; HR, Hazard ratio; HTR6, 5‐Hydroxytryptamine Receptor 6; LNM^pos^, lymph node metastasis‐positive; MAOB, Monoamine Oxidase B; MYL6, Myosin Light Chain 6; OS, overall survival; OSCC, oral squamous cell carcinoma; RORC, RAR Related Orphan Receptor C; Rpl7, Ribosomal Protein L7; SHMT2, Serine Hydroxymethyltransferase 2; siSHMT2a/b, small interfering RNAs a/b targeting SHMT2; STAC3, SH3 And Cysteine Rich Domain 3; TUBB4A, Tubulin Beta 4A Class Iva; WNT5A, Wnt family member 5A; ZNF443, Zinc Finger Protein 443.

## 2 *SHMT2* is a biomarker and therapeutic target in OSCC

Next, we performed a gene expression‐based overall survival (OS) analysis and identified two significant genes, namely Zinc Finger Protein 443 (*ZNF443*) and Serine Hydroxymethyltransferase 2 (*SHMT2*) (Figure 1D, Supplementary Table S2). Specifically, *ZNF443* expression was associated with a reduced risk (Hazard ratio [HR] = 0.238), whereas expression of *SHMT2* (HR = 4.028) suggested a higher risk of mortality. Thus, we further tested their prognostic relevance for OS and recurrence/disease‐free survival (RFS/DFS) in our patient cohort (Figure 1E, Supplementary Figure S2A‐C) as well as in The Cancer Genome Atlas (TCGA) HNSCC dataset (Supplementary Figure S2D‐E) [4]. These analyses indicated that *SHMT2*, but not *ZNF443*, might serve as an OSCC‐specific biomarker for OS. In line with this, *SHMT2* expression was higher in HNSCC tissues compared to normal tissues, as well as in T4 versus T1 tumors of the OSCC subtype, and its expression level increased with higher tumor grade (Supplementary Figure S3). Furthermore, univariate Cox regression analysis demonstrated a significant association of age, T‐stage, N‐stage, and *SHMT2* expression (Relative Risk [RR] = 1.548, *P* = 0.049; 95% confidence interval [CI] = 1.000‐2.396]) with OS in OSCC patients. Multivariate analysis further confirmed the association of *SHMT2* (RR = 1.616, *P* = 0.041; 95% CI = 1.020‐2.559]) (Supplementary Table S3). Intriguingly, downregulating *SHMT2* in SAS, Cal33, and XF354 cells or blocking its activity using an inhibitor [5] reduced proliferation and viability while inducing apoptosis (Figure 1F, Supplementary Figure S4). These data confirmed previous studies and underscored the therapeutic potential of *SHMT2* in OSCC [6, 7].

## 3 Identification of metastasis‐associated genes as potential therapeutic targets

Next, we aimed to characterize the metastasis‐associated transcriptome in our OSCC cohort. First, we compared the transcriptome of primary tumors and matched LNM from each patient. This analysis identified 1,710 deregulated protein‐coding and 990 long non‐coding RNA (lncRNA) genes (false discovery rate [FDR] ≤ 0.05; | log2(Fold change) | ≥ 1). Subsequent gene set enrichment analysis revealed 32 gene sets, including Kirsten Rat Sarcoma Viral Oncogene Homology (*KRAS*) signaling and epithelial‐to‐mesenchymal transition (EMT)‐promoting gene sets, among others, which showed positive enrichment in metastasis (Supplementary Figure S5A‐C, Supplementary Tables S4‐S5). In order to narrow down the list of putative metastasis‐associated genes, we compared gene expression patterns between primary tumors with (*n* = 43) and without (*n* = 24) LNM. This complementary approach uncovered 482 deregulated protein‐coding and 190 lncRNA genes (Supplementary Figure S5D‐E, Supplementary Tables S6‐S7). Intriguingly, a total of 31 gene sets were significantly enriched in LNM‐positive (LNM^pos^) tumors, but only EMT‐promoting genes showed consistent positive enrichment in both differential gene expression analysis (Supplementary Figure S5F). To identify individual genes driving and maintaining metastases, we intersected the lists of differentially expressed genes from both analyses (Supplementary Table S8). This revealed a common set of 41 upregulated and 40 downregulated genes. Gene ontology analysis suggested that differentiation‐associated processes were impaired in both LNM and LNM‐positive tumors (Figure 1G). Importantly, a database search using the canSAR knowledgebase [8] identified four consistently upregulated genes that are targetable with approved clinical drugs. However, their cellular and molecular functions as well as their contribution to OSCC metastasis needs to be established using appropriate in vitro and in vivo models.

## 4 Analysis of metastasis‐associated isoform usage

Finally, we extended our gene‐level expression analysis and characterized gene isoform usage in primary tumors and their matched metastasis. We identified hundreds of alternative transcription events that were either enriched or diminished in metastasis (Supplementary Figure S6A). At the individual gene level, this analysis yielded a list of 114 genes with significant isoform switches (Figure 1H, Supplementary Table S9). The most significant isoform switches were observed for Wnt Family Member 5A (*WNT5A*) and Myosin Light Chain 6 (*MYL6*), whereas SH3 And Cysteine Rich Domain 3 (*STAC3*), and RAR Related Orphan Receptor C (*RORC*) showed the highest combined differences in isoform fraction (dIF) values (Figure 1H). We decided to investigate the isoform switch in *WNT5A* in greater detail. In metastases, the canonical isoform (*WNT5‐201*) was more abundant, while the fractions of *WNT5A‐202* (encoding the same WNT5A protein) and *WNT5‐203* (encoding a shorter protein variant) were significantly reduced (Supplementary Figure S6B). We generated overexpression constructs, transduced SAS cells with these variants, and successfully detected all WNT5A proteins at their expected size (Figure 1I). Intriguingly, overexpression of the canonical WNT5A protein only slightly enhanced spheroid growth in SAS cells but strongly increased their invasive capacity (Supplementary Figure S6C‐D). Moreover, comparison of the different WNT5A isoforms revealed no significant differences in growth (Figure 1J). However, cells overexpressing the *WNT5A‐203* (BC construct) isoform exhibited markedly reduced invasive potential in Matrigel compared to cells expressing other WNT5A protein variants (Figure 1K). These findings suggest that inhibiting the canonical *WNT5A* isoform may represent a therapeutic strategy to prevent metastasis in OSCC, consistent with previous reports [9].

In summary, our study contributes to OSCC profiling and target identification efforts [4, 10] in a unique manner. Our carefully selected sample collection included LNM‐negative and LNM^pos^ primary tumors as well as their matched metastases. Furthermore, RNA isolated in this study was subjected to total RNA sequencing upon ribosomal RNA depletion, providing a more unbiased view of the primary and metastatic oral cancer transcriptome. This approach enabled the identification of coding and non‐coding genes, as well as isoforms, associated with OSCC metastasis. However, additional studies are needed (see Supplementary Discussion) to confirm the described associations and validate the clinical relevance of individual candidates in vitro and in vivo.

## AUTHOR CONTRIBUTIONS

The study was conceptualized by Jonas Pyko, Markus Glaß, and Tony Gutschner with input from Monika Hämmerle and Stefan Hüttelmaier. Experiments were performed by Jonas Pyko, Julia Rosemann, Jana Macho, and Sarah Qasem. Patient tumor samples were collected by Matthias Kappler and Alexander W. Eckert. Pathological sample evaluation was performed by Monika Hämmerle. Computational analysis was performed by Markus Glaß and Stefan Hüttelmaier. Analysis of experimental data was done by Jonas Pyko, Markus Glaß, and Tony Gutschner. The manuscript was written by Jonas Pyko, Markus Glaß, and Tony Gutschner with input from all authors. Figures were prepared by Jonas Pyko, Markus Glaß, and Tony Gutschner. All authors have read and agreed to the final version of the manuscript.

## COMPETING INTERESTS

The authors declare that they have no competing interests.

## FUNDING INFORMATION

This study was supported by intramural funding from the Medical Faculty (Wilhelm‐Roux program, FKZ32/19).

## ETHICS APPROVAL AND CONSENT TO PARTICIPATE

Ethical registry 210/19.08.09/10 was obtained from the Ethics Committee of the Medical Faculty of the University Halle. All patients gave written informed consent (Department of Oral and Maxillofacial Plastic Surgery, University of Halle‐Wittenberg, Germany).

## Supporting information



Supporting Information

Supporting Information

## Data Availability

The data underlying this article are available in the manuscript and in its online additional material. The raw sequencing data have been deposited at NCBI GEO (GSE275870; https://www.ncbi.nlm.nih.gov/geo/query/acc.cgi?&acc=GSE275870). Additional materials generated during the current study are available from the corresponding author upon reasonable request.
