# Supplementary material for: Expression profiling of primary and metastatic oral squamous cell carcinoma identifies progression‐associated transcriptome changes and therapeutic vulnerabilities
Source: Cancer Commun (Lond). 2025 Jan 7;45(4):433–7. doi: 10.1002/cac2.12660 (PMC11999880; doi:10.1002/cac2.12660)
Supplement: Supplementary file 1 — Supporting Information [file CAC2-45-433-s002.docx]

**Supplementary Information**

**Expression profiling of primary and metastatic oral squamous cell carcinoma identifies progression-associated transcriptome changes and therapeutic vulnerabilities**

Jonas Pyko^1,*^, Markus Glaß^2,*^, Julia Rosemann^1^, Matthias Kappler^3^, Jana Macho^1^, Sarah Qasem^1,6^, Stefan Hüttelmaier^2^, Alexander W. Eckert^4^, Monika Haemmerle^5^, Tony Gutschner^1,#^

^1^Institute of Molecular Medicine, Section for RNA biology and pathogenesis, Faculty of Medicine, Martin Luther University Halle-Wittenberg, 06120 Halle (Saale), Germany

^2^Institute of Molecular Medicine, Section for Molecular Cell Biology, Faculty of Medicine, Martin Luther University Halle-Wittenberg, 06120 Halle (Saale), Germany

^3^Department of Oral and Maxillofacial Plastic Surgery, Faculty of Medicine, Martin Luther University Halle-Wittenberg, 06120 Halle (Saale), Germany

^4^Department of Cranio Maxillofacial Surgery, Paracelsus Medical University, 90471 Nuremberg, Germany

^5^Institute of Pathology, Section for Experimental Pathology, Faculty of Medicine, Martin Luther University Halle-Wittenberg, 06120 Halle (Saale), Germany

^6^Current address: Abbvie Deutschland GmbH & Co. KG, 67061 Ludwigshafen am Rhein, Germany

***Jonas Pyko and Markus Glaß contributed equally to this work.**

**^#^Corresponding author:**

Tony Gutschner, Institute of Molecular Medicine, Section for RNA biology and pathogenesis, Faculty of Medicine, Martin Luther University Halle-Wittenberg, Kurt-Mothes-Str. 3a, 06120 Halle (Saale), Germany, Email: tony.gutschner@uk-halle.de, Tel.: +49-345-557-3945

**Supplementary Materials and Methods**

**Patient cohort and sample preparation**

72 patients diagnosed with OSCC who provided written informed consent were included in this study. The primary tumors originated from the mucosa of the jaw, oral cavity, or tongue. The median age at diagnosis was 58.9 years for male patients (*n* = 52) and 70 years for female patients (*n* = 20). Survival data were available from 69 patients, enabling us to calculate a median survival time of 24.5 months and 5- and 10-year survival rates of 33% and 17%, respectively. Sex had no influence on the survival rate (data not shown).

Tumor and lymph node metastasis tissues were snap frozen in liquid nitrogen immediately after surgical resection. Cryosectioning was performed to obtain tissue slices for hematoxylin and eosin staining to determine tumor content, as well as material for RNA isolation. Primary tumor samples with at least 60% tumor content were further processed, yielding a total of 87 primary tumors as well as 21 metastases (15 - 95% tumor content) from which total RNA was isolated and subjected to RNA sequencing. For our transcriptome analysis, RNA was isolated from one primary tumor per patient (*n* = 59), while 13 patients had tumors large enough to allow multi-regional sampling (11 patients with two samples, one patient with three, and one patient with four primary tumor samples), resulting in a total of 87 primary tumor samples from 71 individual patients. Importantly, 20 matched lymph node metastases (LNM) from 11 patients (up to three metastases per patient), as well as one LNM from an additional patient without available primary tumor material, were included, allowing a direct comparison between gene expression programs in primary tumors and LNM. Moreover, 24 patients were diagnosed with lymph node-negative (N0, LNM^neg^) OSCC, while 12, 28, and 3 patients had positive lymph node status (i.e. N1, N2, and N3, respectively). For the remaining 5 patients, the N status was unknown.

**Cell culture**

Human OSCC cells SAS, Cal33, and XF354, a kind gift from Prof. Dr. Daniel Zips, were cultured at 37°C and 5% CO_2_ in Roswell Park Memorial Institute (RPMI)-1640 medium supplemented with 10% fetal bovine serum (FBS), 100 Units/mL penicillin, 100 µg/ml streptomycin, 2 mmol/L L-glutamine (all from Thermo Fisher Scientific, Waltham, MA, USA), and 1 mmol/L sodium pyruvate (Sigma Life Science, St. Louis, MO, USA). For the production of lentiviral particles, HEK293T cells (ACC 635, DSMZ, Braunschweig, Germany, [1]) were cultured in Dulbecco’s Modified Eagle Medium (DMEM, Thermo Fisher Scientific, Waltham, MA, USA) supplemented with 10% FBS. Regular testing for mycoplasma contamination was conducted.

**Cloning**

For doxycycline (Dox)-inducible overexpression of the different *WNT5A* isoforms, a modified pCW-CRISPR associated protein 9 (Cas9) plasmid (a kind gift from Eric Lander & David Sabatini, Addgene plasmid # 50661 [2]) was used. Cas9 was replaced by an N-terminal FLAG/hemagglutinin (HA)-tag followed by a multiple cloning site (MCS). Plasmid pcDNA-WNT5A (Addgene plasmid #35911 [3]) served as the cloning template. PCR products were digested with NheI and XhoI (Thermo Fisher Scientific, Waltham, MA, USA) and inserted into the MCS. The relevant cloning primers are listed in **Supplementary Table S10**. The following primer combinations were used for cloning the respective isoform-encoded proteins: Primers A+D (canonical Wnt5a, encoded by WNT5A-201/202); Primers A+C (no known isoform); Primers B+D (N-terminal truncated WNT5A, encoded by WNT5A-205); and Primers B+C (N- and C-terminal truncated WNT5A, encoded by WNT5A-203). The AlphaFold Protein Structure Database [4, 5] and UniProt [6] were used to predict the structure of the WNT5A variants shown in **Figure 1I**.

**Lentiviral transduction and WNT5A isoform overexpression**

The pCW-FLAG/HA-MCS (empty vector, EV) or pCW-FLAG/HA-WNT5A isoform-encoding plasmids were transfected into HEK293T cells along with the packaging vector psPAX2 and the envelope expressing vector pMD2.G (gifts from Didier Trono, Addgene plasmids #12260 and #12259, respectively) using Turbofect (Thermo Fisher Scientific, Waltham, MA, USA) according to the manufacturer’s instructions. Lentiviral particles were collected after 72 hours and filtered through a 0.45 µm membrane (TPP, Trasadingen, Switzerland). For transduction, cells were mixed with fresh lentiviral stocks and allowed to attach. For subsequent antibiotic selection of transduced SAS cells, puromycin (1.5 µg/mL; Thermo Fisher Scientific, Waltham, MA, USA) was added, which resulted in complete cell death of non-transduced control cells within 48 - 72 hours. *WNT5A* isoform expression in stably transduced cells was induced by addition of 1 µg/mL Doxycycline (Sigma Life Science, St. Louis, MO, USA).

**Transfection of small interfering RNAs (siRNAs)**

Transfection of siRNAs was done as described previously [7]. Briefly, Opti-MEM (Thermo Fisher Scientific, Waltham, MA, USA) was used to dissolve, mix, and incubate the transfection reagent (RNAiMax, Thermo Fisher Scientific, Waltham, MA, USA) and siRNAs for 5 minutes. The transfection mix was pipetted into 6-well plates, and 6×10^5^ cells (in antibiotics-free medium) were added, yielding a final siRNA concentration of 15 nmol/L. The following siRNAs were used: siSHMT2a (5’-AGACCGAAGUGCCAUCACAdTdT-3’) and siSHMT2b (5’- CGAGGCUACUCACUGGUAUdTdT-3’). Non-targeting siRNAs (siAllStars Negative Control) from Qiagen (Hilden, Germany) were used as transfection control.

**Bromodeoxyuridine (BrdU) assay**

BrdU assay was conducted using a Cell Proliferation ELISA BrdU kit (Roche, Basel, Switzerland). 24 hours after siRNA transfection, 3×10^3^ SAS cells or 5×10^3^ Cal33 and XF354 cells, respectively, were seeded in 96-well plates and incubated overnight. The BrdU labeling solution was added for another 24 hours. Staining was performed according to the manufacture’s protocol.

**Viability assay**

For siRNA-mediated knockdown experiments, 5×10^3^ cells were seeded 24 hours after transfection in 96-well plates. 96 hours after transfection, cells were incubated with 1 mg/ml of 3-(4,5-Di-2-yl)-2,5-ditetrazolium bromide (MTT, Merck, Darmstadt, Germany) solution for two hours. Subsequently, the precipitate was dissolved in DMSO (Carl Roth, Karlsruhe, Germany), and absorbance was measured at 565 nm using a Tecan Infinite 200 Pro plate reader (Tecan, Männedorf, Switzerland). For inhibitor treatment, 1×10^3^ SAS, 2×10^3^ Cal33, and 5×10^3^ XF354 cells were seeded in 96-well plates in medium containing the respective concentration of the SHMT1/2-specific inhibitor SHIN1/RZ-2994 (MedchemExpress, Monmouth Junction, NJ, USA) or DMSO. Measurements were conducted after 96 hours of drug treatment.

**Two-dimensional cell proliferation analysis**

A total of 1×10^3^ cells were seeded 24 hours after siRNA transfection in 96-well plates. The plates were transferred to an IncuCyte® S3 Live Cell Analysis System (Sartorius, Göttingen, Germany), and cells were allowed to proliferate for 120 hours. Images were captured every 6 hours, and confluence was calculated using the IncuCyte software.

**Three-dimensional sphere growth and matrigel-based invasion assay**

A total of 1×10^3^ cells in 50 µl complete medium were seeded into an ultralow attachment 96-well plate (Corning, Corning, NY, USA). Cells were centrifuged at 300 g for 5 minutes and allowed to form spheres overnight. For three-dimensional growth, an additional 150 µl of complete media was added. For matrigel-based invasion assays, spheres were embedded in 50 µl of cold Matrigel (Corning, Corning, NY, USA), centrifuged at 300 g for 6 minutes at 4°C, and incubated for 30 minutes at 37°C. An additional 100 µl of complete medium per well (total volume = 200 µl) was added. Images were captured immediately after this incubation and afterwards every 6 hours over a 48-hour period using an IncuCyte S3 Live Cell Analysis System (Sartorius, Göttingen, Germany).

**RNA isolation and total RNA-Sequencing**

Total RNA from both patient-derived and cell culture-derived samples was isolated using the acid guanidinium thiocyanate-phenol-chloroform (AGPC) method [8]. The incubation time with the AGPC solution for patient-derived tumour samples was extended to 10 minutes, and the samples were resuspended at room temperature. For total RNA sequencing, 1 µg of tumor RNA was used for sequencing library preparation based on ribosome depletion, performed by Genewiz (Leipzig, Germany). Strand-specific, paired-end sequencing (2x 150 bp) was performed by Genewiz on a NovaSeq platform (Illumina, San Diego, CA, USA), yielding between 2 x 18 million and 2 x 116 million sequencing reads (avg. 2 x 62 million reads per sample).

**Differential gene expression analysis**

For RNA sequencing data analyses, adapter sequences as well as low-quality read ends were trimmed using Cutadapt (v 2.8) [9]. The processed sequencing reads were aligned to the human reference genome (UCSC hg38) using HiSat2 (v 2.1.0) [10]. Samtools (v 1.10; [11]) was used to extract primary alignments and index the resulting BAM files. FeatureCounts (v 2.0.0; [12]) was used for summarizing gene-mapped reads. ENSEMBL (GRCmh38 v100; [13]) was used as the annotation basis. Differential gene expression was determined using the R package edgeR (v 3.42.4), which employs trimmed mean of M-values normalization [14, 15]. Either the exactTest function or a generalized linear model (glmFit - used for comparisons between matched tumor and metastasis samples) was applied. A false discovery rate (FDR) value below 0.05, combined with | log2(Fold change) | ≥ 1, was considered as a significance threshold for differential gene expression analysis.

**Unsupervised clustering**

Unsupervised clustering was performed using the R package ConsensusClusterPlus (v1.64.0; [16]). Median-centered Fragments Per Kilobase Million (FPKM) values of the 2,500 most variable protein-coding and non-coding RNA genes, measured by median absolute deviation, were used for clustering. The agglomerative hierarchical clustering algorithm, based on Pearson correlation distances, was used with 80% item resampling, 100% gene resampling, a maximum evaluated k of 10 and 1,000 resampling steps.

**Gene set enrichment analysis**

Gene set enrichment analysis (GSEA) was performed using the R package clusterProfiler (v 4.8.2; [17]) and MSigDB gene sets (v2023.1) [18], utilizing the fgsea algorithm with the exponent parameter set to 0 for unweighted analyses of log2 fold-change sorted gene lists obtained from differential gene expression analyses. The STRING database was used to perform a gene set overrepresentation test [19].

**Differential isoform usage**

Transcript abundances were quantified using Salmon (v1.5.2; [20]) in mapping mode and ENSEMBL (GRCmh38 v100) [13], for annotation. Isoform switches between primary tumor and matched lymph node metastases were determined using the R package IsoformSwitchAnalyzeR (v2.0.1) and the DEXseq algorithm to test for isoform switches [21, 22]. Specifically, we applied IsoformSwitchAnalyzeR to characterize metastasis-associated isoform usage and identify significant isoform switches [22]. The tool measured relative isoform usage as the fraction of gene expression originating from each associated isoform, i.e. isoform fraction (IF) for each condition (primary tumor or LNM) and calculated the differences in IF values (dIF) between conditions.

**Survival analysis**

Overall survival probabilities were calculated using the log-rank test implemented in the R package survival (function survdiff, setting rho = 0) [23]. Gene expression-based survival analyses were performed using the mean expression values of all primary tumor samples per patient. Only genes with at least 10 samples per group (high vs. low expression) were considered. To evaluate the prognostic relevance of *ZNF443* and *SHMT2* in an independent patient cohort, we leveraged the cBioPortal (https://www.cbioportal.org/) [24-26] and performed survival analyses using the TCGA HNSCC dataset (*n* = 520 samples). Filtering for anatomical subsites (alveolar ridge, buccal mucosa, floor of mouth, hard palate, lip, oral cavity, and tongue) allowed us to focus specifically on OSCC samples (*n* = 315).

**Western blotting**

A total of 8×10^5^ cells were seeded in 6 cm plates. Cells were gently harvested, washed in PBS, and lysed in total lysis buffer (50 mmol/L Tris(hydroxymethyl)aminomethane (Tris), 50 mmol/L NaCl, 2 mmol/L MgCl2, 1% (w/v) SDS) supplemented with protease inhibitor cocktail (Roche, Basel, Switzerland). Following SDS-Polyacrylamid gel electrophoresis and wet blotting to transfer proteins onto a nitrocellulose membrane (Th.Geyer, Renningen, Germany), individual proteins of interest were detected using the following primary antibodies: Rpl7 (A300-741A, Bethyl Laboratories, Montgomery, TX, USA), Shmt2 (#33443S), Parp (#9532S), β-actin (#8457S), Wnt5a/b (#2530S, all Cell Signaling Technologies, Danvers, MA, USA). The following secondary antibodies were used respectively: IRDye® 800CW Donkey anti-Rabbit or IRDye® 680RD Donkey anti-Rabbit (all from LI-COR Biosciences, Lincoln, NE, USA). Protein expression levels were evaluated using an Odyssey infrared scanner (LI-COR).

**Statistical analysis**

Experiments were repeated at least three times. Unless stated otherwise, data are represented as mean with standard deviation. Statistical tests used for experimental data included Student’s t-test (comparing two means), one-way ANOVA (multiple comparisons), and two-way ANOVA (grouped data), all of which were corrected using Holm-Sidak’s test. GraphPad Prism 8.0 (GraphPad Software, San Diego, CA, USA) was used to conduct statistical tests. Differences with *P* ≤ 0.05 were considered significant (**P* ≤ 0.05; ***P* ≤ 0.01; ****P* ≤ 0.001). The Cox regression hazard model for analysis of overall survival was performed using SPSS 28.0 (IBM, Armonk, NY, USA).

**Supplementary Discussion**

Oral squamous cell carcinoma, a major subgroup of head and neck squamous cell carcinoma (HNSCC), is an aggressive disease that preferentially spreads to cervical lymph nodes. Positive lymph node status is associated with poor prognosis and is an important predictor of disease-specific and overall survival in OSCC [27-29]. Hence, a better understanding of the molecular mechanisms underlying oral cancer metastasis, and the identification of therapeutic vulnerabilities, is crucial for preventing and treating advanced-stage disease. To achieve this goal, several studies have been performed in the past to identify gene expression signatures that predict patients’ survival and the occurrence of metastases in HNSCC, particularly OSCC [30-36]. Some of these studies leveraged public clinical data and bulk RNA-seq data provided by the TCGA [37-39]. In addition, single-cell RNA-seq profiling has been performed on a small OSCC cohort, and some studies have integrated these data with bulk RNA-seq data to identify biomarkers and develop expression-based risk models [40, 41]. Our study adds to these existing efforts in a unique manner, as our carefully selected sample collection included lymph node metastasis-negative (LNM^neg^) and lymph node metastasis-positive (LNM^pos^) primary tumors as well as their matched metastases. Furthermore, unlike microarray-based expression profiling or sequencing of polyadenylated RNA used in previous studies, RNA isolated in this study was subjected to total RNA-seq upon ribosomal RNA depletion, providing a more unbiased view of the primary and metastatic oral cancer transcriptome. Importantly, the integration of expression data with clinical data revealed several interesting candidate genes with potential prognostic and therapeutic relevance due to their role in OSCC development and progression. For example, we identified *SHMT2* as a marker for poor survival and demonstrated that genetic or chemical inhibition of this metabolic enzyme impaired oral cancer cell proliferation and induced apoptosis *in vitro*. SHMT2 catalyses the conversion of serine to glycine, generating the major source of activated one-carbon units for *de novo* nucleotide biosynthesis in mitochondria. Consequently, overexpression of *SHMT2* promoted cell proliferation and has been observed in several human cancers [42-44]. Our data presented herein confirmed previous studies that show an increased expression of *SHMT2* and its prognostic association with patient survival and tumor characteristics in head and neck cancers [45, 46]. Importantly, the therapeutic potential of *SHMT2* in oral cancer has also been demonstrated by other groups, and small molecule inhibitors for *in vitro* and *in vivo* applications have been developed [44, 45, 47-50]. Future studies should therefore try to evaluate these findings in pre-clinical studies, such as xenograft assays and genetic mouse models of OSCC [51]. Potential synergistic combinations between Shmt2 inhibitors and other drugs like Methotrexate or inhibitors of the G2/M checkpoint, which have shown promising results in leukaemia models, should also be considered [48, 52].

In addition to *SHMT2*, we identified several other druggable candidates relevant to OSCC metastasis. We highlighted *TUBB4A*, *GABRG3*, *MAOB*, and *HTR6*, because they have approved clinical drugs already available, and some studies have linked them to prognosis and metastasis in breast and prostate cancer, as well as osteosarcoma [53-57]. For example, *TUBB4A* was recently shown to protect the nucleus from genomic instability during prostate cancer cell migration. Moreover, *TUBB4A* overexpression promoted cancer progression, whereas its deletion reduced tumor growth and metastasis [53]. In the future, the function of *TUBB4A* in oral cancer should be investigated in greater detail. To assess its therapeutic potential, microtubule targeting agents like eribulin, vinorelbine, and others could be directly tested *in vitro* and *in vivo*. Alternatively, antibody-drug conjugates (ADCs), such as Tisotumab vedotin, RN765C, or anti-tissue factor ADCs, could be explored to specifically deliver microtubule inhibitors to oral cancer cells [58-60]. In addition to the four genes mentioned above, several other metastasis-associated genes were identified in this study, some of which possess enzymatic activities and/or could be targeted by chemical compounds. Therefore, the cellular and molecular functions of these genes should be analysed in detail, and further studies, including larger patient cohorts, are necessary to validate their relevance in cancer metastasis. Confirmed drivers of disease could then be targeted through the development of direct enzymatic inhibitors, proteolysis-targeting chimeras, or other targeted protein degradation approaches [61-63].

Last but not least, we extended our gene-level expression analysis and characterized the differential usage of gene isoforms in primary tumors and their matched metastasis. Isoform switches can have significant functional consequences and are especially prominent in human cancers [64-68]. Here, we identified *WNT5A* isoform switches that favoured the expression of a canonical, invasion-promoting isoform, *WNT5A-201*. This pro-oncogenic role of *WNT5A* in head and neck cancer has been identified by others as well [69-72]. However, cancer-specific functions of *WNT5A* as either an oncogene or a tumor suppressor have been reported, and different *WNT5A* isoforms have been shown to exert opposite roles in cancer cell growth [73]. Our data did not reveal a significant impact of the canonical *WNT5A* isoform on three-dimensional cell growth. However, inhibition of the secreted WNT5A protein by neutralizing antibodies or alternative approaches might be a promising therapeutic strategy to inhibit the metastatic cascade. This potential should be tested experimentally using appropriate *in vivo* models. In addition to *WNT5A*, we identified isoform switches in several other genes. For example, we discovered increased expression of *MYL6-207* accompanied by a simultaneous reduction in *MYL6-218*. Importantly, *MYL6-218* and *MYL6-207* have been shown to be differentially expressed in tonic and phasic smooth muscle, with both isoforms proposed to influence myosin ATPase activity and shortening velocity [74, 75]. However, the isoform-specific functions of *MYL6* in cancer remain elusive. Our data imply a potential role in cell migration and metastasis, which warrants further investigation.

Overall, our data underscore the importance of isoform-resolved expression analyses and functional interrogation to gain a deeper understanding of the molecular mechanisms driving cancer progression. Incorporating isoform-specific expression analyses in biomarker studies and drug development efforts could enhance marker sensitivity and specificity as well as drug efficiency and safety.

**Supplementary references**

1. DuBridge RB, Tang P, Hsia HC, Leong PM, Miller JH, Calos MP. Analysis of mutation in human cells by using an Epstein-Barr virus shuttle system. Mol Cell Biol. 1987;7(1):379-87.

2. Wang T, Wei JJ, Sabatini DM, Lander ES. Genetic screens in human cells using the CRISPR-Cas9 system. Science. 2014;343(6166):80-4.

3. Najdi R, Proffitt K, Sprowl S, Kaur S, Yu J, Covey TM, et al. A uniform human Wnt expression library reveals a shared secretory pathway and unique signaling activities. Differentiation. 2012;84(2):203-13.

4. Jumper J, Evans R, Pritzel A, Green T, Figurnov M, Ronneberger O, et al. Highly accurate protein structure prediction with AlphaFold. Nature. 2021;596(7873):583-9.

5. Varadi M, Anyango S, Deshpande M, Nair S, Natassia C, Yordanova G, et al. AlphaFold Protein Structure Database: massively expanding the structural coverage of protein-sequence space with high-accuracy models. Nucleic Acids Res. 2022;50(D1):D439-D44.

6. UniProt C. UniProt: the Universal Protein Knowledgebase in 2023. Nucleic Acids Res. 2023;51(D1):D523-D31.

7. Rosemann J, Pyko J, Jacob R, Macho J, Kappler M, Eckert AW, et al. NANOS1 restricts oral cancer cell motility and TGF-ss signaling. Eur J Cell Biol. 2024;103(2):151400.

8. Chomczynski P, Sacchi N. Single-step method of RNA isolation by acid guanidinium thiocyanate-phenol-chloroform extraction. Anal Biochem. 1987;162(1):156-9.

9. Martin M. Cutadapt removes adapter sequences from high-throughput sequencing reads. 2011. 2011;17(1):3.

10. Kim D, Langmead B, Salzberg SL. HISAT: a fast spliced aligner with low memory requirements. Nat Methods. 2015;12(4):357-60.

11. Li H, Handsaker B, Wysoker A, Fennell T, Ruan J, Homer N, et al. The Sequence Alignment/Map format and SAMtools. Bioinformatics. 2009;25(16):2078-9.

12. Liao Y, Smyth GK, Shi W. featureCounts: an efficient general purpose program for assigning sequence reads to genomic features. Bioinformatics. 2014;30(7):923-30.

13. Aken BL, Achuthan P, Akanni W, Amode MR, Bernsdorff F, Bhai J, et al. Ensembl 2017. Nucleic Acids Res. 2017;45(D1):D635-D42.

14. Robinson MD, McCarthy DJ, Smyth GK. edgeR: a Bioconductor package for differential expression analysis of digital gene expression data. Bioinformatics. 2010;26(1):139-40.

15. Robinson MD, Oshlack A. A scaling normalization method for differential expression analysis of RNA-seq data. Genome Biol. 2010;11(3):R25.

16. Wilkerson MD, Hayes DN. ConsensusClusterPlus: a class discovery tool with confidence assessments and item tracking. Bioinformatics. 2010;26(12):1572-3.

17. Yu G, Wang LG, Han Y, He QY. clusterProfiler: an R package for comparing biological themes among gene clusters. OMICS. 2012;16(5):284-7.

18. Liberzon A, Subramanian A, Pinchback R, Thorvaldsdottir H, Tamayo P, Mesirov JP. Molecular signatures database (MSigDB) 3.0. Bioinformatics. 2011;27(12):1739-40.

19. Szklarczyk D, Kirsch R, Koutrouli M, Nastou K, Mehryary F, Hachilif R, et al. The STRING database in 2023: protein-protein association networks and functional enrichment analyses for any sequenced genome of interest. Nucleic Acids Res. 2023;51(D1):D638-D46.

20. Patro R, Duggal G, Love MI, Irizarry RA, Kingsford C. Salmon provides fast and bias-aware quantification of transcript expression. Nat Methods. 2017;14(4):417-9.

21. Anders S, Reyes A, Huber W. Detecting differential usage of exons from RNA-seq data. Genome Res. 2012;22(10):2008-17.

22. Vitting-Seerup K, Sandelin A. The Landscape of Isoform Switches in Human Cancers. Mol Cancer Res. 2017;15(9):1206-20.

23. Therneau TM, Grambsch PM. Modeling Survival Data: Extending the Cox Model. 1 ed. Gail MH, Samet JM, editors. New York: Springer New York, NY; 2000. XIV, 350 p.

24. Cerami E, Gao J, Dogrusoz U, Gross BE, Sumer SO, Aksoy BA, et al. The cBio cancer genomics portal: an open platform for exploring multidimensional cancer genomics data. Cancer Discov. 2012;2(5):401-4.

25. de Bruijn I, Kundra R, Mastrogiacomo B, Tran TN, Sikina L, Mazor T, et al. Analysis and Visualization of Longitudinal Genomic and Clinical Data from the AACR Project GENIE Biopharma Collaborative in cBioPortal. Cancer Res. 2023;83(23):3861-7.

26. Gao J, Aksoy BA, Dogrusoz U, Dresdner G, Gross B, Sumer SO, et al. Integrative analysis of complex cancer genomics and clinical profiles using the cBioPortal. Sci Signal. 2013;6(269):pl1.

27. Lee H, Roh JL, Cho KJ, Choi SH, Nam SY, Kim SY. Number of positive lymph nodes better predicts survival for oral cavity cancer. J Surg Oncol. 2019;119(6):675-82.

28. Shah JP. Cervical lymph node metastases--diagnostic, therapeutic, and prognostic implications. Oncology (Williston Park). 1990;4(10):61-9; discussion 72, 6.

29. Zanoni DK, Montero PH, Migliacci JC, Shah JP, Wong RJ, Ganly I, et al. Survival outcomes after treatment of cancer of the oral cavity (1985-2015). Oral Oncol. 2019;90:115-21.

30. Alfieri S, Carenzo A, Platini F, Serafini MS, Perrone F, Galbiati D, et al. Tumor Biomarkers for the Prediction of Distant Metastasis in Head and Neck Squamous Cell Carcinoma. Cancers (Basel). 2020;12(4).

31. Goesswein D, Habtemichael N, Gerhold-Ay A, Mazur J, Wunsch D, Knauer SK, et al. Expressional analysis of disease-relevant signalling-pathways in primary tumours and metastasis of head and neck cancers. Sci Rep. 2018;8(1):7326.

32. Nguyen ST, Hasegawa S, Tsuda H, Tomioka H, Ushijima M, Noda M, et al. Identification of a predictive gene expression signature of cervical lymph node metastasis in oral squamous cell carcinoma. Cancer Sci. 2007;98(5):740-6.

33. Qiao B, Zhao M, Wu J, Wu H, Zhao Y, Meng F, et al. A Novel RNA-Seq-Based Model for Preoperative Prediction of Lymph Node Metastasis in Oral Squamous Cell Carcinoma. Biomed Res Int. 2020;2020:4252580.

34. Roepman P, Wessels LF, Kettelarij N, Kemmeren P, Miles AJ, Lijnzaad P, et al. An expression profile for diagnosis of lymph node metastases from primary head and neck squamous cell carcinomas. Nat Genet. 2005;37(2):182-6.

35. Watanabe H, Mogushi K, Miura M, Yoshimura R, Kurabayashi T, Shibuya H, et al. Prediction of lymphatic metastasis based on gene expression profile analysis after brachytherapy for early-stage oral tongue carcinoma. Radiother Oncol. 2008;87(2):237-42.

36. Zhang Y, Luo X, Yu J, Qian K, Zhu H. An Immune Feature-Based, Three-Gene Scoring System for Prognostic Prediction of Head-and-Neck Squamous Cell Carcinoma. Front Oncol. 2021;11:739182.

37. Cancer Genome Atlas N. Comprehensive genomic characterization of head and neck squamous cell carcinomas. Nature. 2015;517(7536):576-82.

38. Chen X, Zhang Y, Chen S, Yang Y, Sun G, Pan P. Construction of a nomogram for predicting HNSCC distant metastasis and identification of EIF5A as a hub gene. Sci Rep. 2024;14(1):13367.

39. Zhu H, Bao Y, Dou X, Zuo X, Ye J, Ma H, et al. KIF2C is a critical regulator for malignant progression of head and neck squamous cell carcinoma. Am J Cancer Res. 2024;14(5):2538-54.

40. Han PZ, Tan LC, Ouyang QS, Yu PC, Shi X, Hu JQ, et al. Development and validation of a gene model predicting lymph node metastasis and prognosis of oral squamous cell carcinoma based on single-cell and bulk RNA-seq analysis. J Oral Pathol Med. 2023;52(5):389-401.

41. Puram SV, Tirosh I, Parikh AS, Patel AP, Yizhak K, Gillespie S, et al. Single-Cell Transcriptomic Analysis of Primary and Metastatic Tumor Ecosystems in Head and Neck Cancer. Cell. 2017;171(7):1611-24 e24.

42. Xie M, Pei DS. Serine hydroxymethyltransferase 2: a novel target for human cancer therapy. Invest New Drugs. 2021;39(6):1671-81.

43. Zeng Y, Zhang J, Xu M, Chen F, Zi R, Yue J, et al. Roles of Mitochondrial Serine Hydroxymethyltransferase 2 (SHMT2) in Human Carcinogenesis. J Cancer. 2021;12(19):5888-94.

44. Zhao LN, Bjorklund M, Caldez MJ, Zheng J, Kaldis P. Therapeutic targeting of the mitochondrial one-carbon pathway: perspectives, pitfalls, and potential. Oncogene. 2021;40(13):2339-54.

45. Jin Y, Jung SN, Lim MA, Oh C, Piao Y, Kim HJ, et al. SHMT2 Induces Stemness and Progression of Head and Neck Cancer. Int J Mol Sci. 2022;23(17).

46. Wu ZZ, Wang S, Yang QC, Wang XL, Yang LL, Liu B, et al. Increased Expression of SHMT2 Is Associated With Poor Prognosis and Advanced Pathological Grade in Oral Squamous Cell Carcinoma. Front Oncol. 2020;10:588530.

47. Ducker GS, Ghergurovich JM, Mainolfi N, Suri V, Jeong SK, Hsin-Jung Li S, et al. Human SHMT inhibitors reveal defective glycine import as a targetable metabolic vulnerability of diffuse large B-cell lymphoma. Proc Natl Acad Sci U S A. 2017;114(43):11404-9.

48. Garcia-Canaveras JC, Lancho O, Ducker GS, Ghergurovich JM, Xu X, da Silva-Diz V, et al. SHMT inhibition is effective and synergizes with methotrexate in T-cell acute lymphoblastic leukemia. Leukemia. 2021;35(2):377-88.

49. Liao Y, Wang F, Zhang Y, Cai H, Song F, Hou J. Silencing SHMT2 inhibits the progression of tongue squamous cell carcinoma through cell cycle regulation. Cancer Cell Int. 2021;21(1):220.

50. Zhang H, Che Y, Xuan B, Wu X, Li H. Serine hydroxymethyltransferase 2 (SHMT2) potentiates the aggressive process of oral squamous cell carcinoma by binding to interleukin enhancer-binding factor 2 (ILF2). Bioengineered. 2022;13(4):8785-97.

51. Lee YM, Hsu CL, Chen YH, Ou DL, Hsu C, Tan CT. Genomic and Transcriptomic Landscape of an Oral Squamous Cell Carcinoma Mouse Model for Immunotherapy. Cancer Immunol Res. 2023;11(11):1553-67.

52. Pikman Y, Ocasio-Martinez N, Alexe G, Dimitrov B, Kitara S, Diehl FF, et al. Targeting serine hydroxymethyltransferases 1 and 2 for T-cell acute lymphoblastic leukemia therapy. Leukemia. 2022;36(2):348-60.

53. Gao S, Wang S, Zhao Z, Zhang C, Liu Z, Ye P, et al. TUBB4A interacts with MYH9 to protect the nucleus during cell migration and promotes prostate cancer via GSK3beta/beta-catenin signalling. Nat Commun. 2022;13(1):2792.

54. Huang HC, Hsieh YH, Hsiao CH, Lin CY, Wang SS, Ho KH, et al. MAOB expression correlates with a favourable prognosis in prostate cancer, and its genetic variants are associated with the metastasis of the disease. J Cell Mol Med. 2024;28(8):e18229.

55. Ma T, Peng C, Wu D, Yang S, Ji L, Cheng Z, et al. Immune-based prognostic biomarkers associated with metastasis of osteosarcoma. Gen Physiol Biophys. 2023;42(1):1-12.

56. Sousa DM, Fernandes V, Lourenco C, Carvalho-Maia C, Estevao-Pereira H, Lobo J, et al. Profiling the Adrenergic System in Breast Cancer and the Development of Metastasis. Cancers (Basel). 2022;14(22).

57. Zhan D, Wang X, Zheng Y, Wang S, Yang B, Pan B, et al. Integrative dissection of 5-hydroxytryptamine receptors-related signature in the prognosis and immune microenvironment of breast cancer. Front Oncol. 2023;13:1147189.

58. de Bono JS, Concin N, Hong DS, Thistlethwaite FC, Machiels JP, Arkenau HT, et al. Tisotumab vedotin in patients with advanced or metastatic solid tumours (InnovaTV 201): a first-in-human, multicentre, phase 1-2 trial. Lancet Oncol. 2019;20(3):383-93.

59. Theunissen JW, Cai AG, Bhatti MM, Cooper AB, Avery AD, Dorfman R, et al. Treating Tissue Factor-Positive Cancers with Antibody-Drug Conjugates That Do Not Affect Blood Clotting. Mol Cancer Ther. 2018;17(11):2412-26.

60. Wong OK, Tran TT, Ho WH, Casas MG, Au M, Bateman M, et al. RN765C, a low affinity EGFR antibody drug conjugate with potent anti-tumor activity in preclinical solid tumor models. Oncotarget. 2018;9(71):33446-58.

61. Bekes M, Langley DR, Crews CM. PROTAC targeted protein degraders: the past is prologue. Nat Rev Drug Discov. 2022;21(3):181-200.

62. Ibrahim HS, Sippl W. Editorial: Application of PROTACs as a Novel Strategy for Drug Discovery. Front Chem. 2021;9:740196.

63. Zhao L, Zhao J, Zhong K, Tong A, Jia D. Targeted protein degradation: mechanisms, strategies and application. Signal Transduct Target Ther. 2022;7(1):113.

64. Davuluri RV, Suzuki Y, Sugano S, Plass C, Huang TH. The functional consequences of alternative promoter use in mammalian genomes. Trends Genet. 2008;24(4):167-77.

65. Kahles A, Lehmann KV, Toussaint NC, Huser M, Stark SG, Sachsenberg T, et al. Comprehensive Analysis of Alternative Splicing Across Tumors from 8,705 Patients. Cancer Cell. 2018;34(2):211-24 e6.

66. Thomas JD, Polaski JT, Feng Q, De Neef EJ, Hoppe ER, McSharry MV, et al. RNA isoform screens uncover the essentiality and tumor-suppressor activity of ultraconserved poison exons. Nat Genet. 2020;52(1):84-94.

67. Zhang Y, Yan L, Zeng J, Zhou H, Liu H, Yu G, et al. Pan-cancer analysis of clinical relevance of alternative splicing events in 31 human cancers. Oncogene. 2019;38(40):6678-95.

68. Zhang Y, Yao X, Zhou H, Wu X, Tian J, Zeng J, et al. OncoSplicing: an updated database for clinically relevant alternative splicing in 33 human cancers. Nucleic Acids Res. 2022;50(D1):D1340-D7.

69. Chen C, Luo L, Xu C, Yang X, Liu T, Luo J, et al. Tumor specificity of WNT ligands and receptors reveals universal squamous cell carcinoma oncogenes. BMC Cancer. 2022;22(1):790.

70. Khan W, Haragannavar VC, Rao RS, Prasad K, Sowmya SV, Augustine D, et al. P-Cadherin and WNT5A expression in assessment of lymph node metastasis in oral squamous cell carcinoma. Clin Oral Investig. 2022;26(1):259-73.

71. Kumawat K, Gosens R. WNT-5A: signaling and functions in health and disease. Cell Mol Life Sci. 2016;73(3):567-87.

72. Prgomet Z, Axelsson L, Lindberg P, Andersson T. Migration and invasion of oral squamous carcinoma cells is promoted by WNT5A, a regulator of cancer progression. J Oral Pathol Med. 2015;44(10):776-84.

73. Bauer M, Benard J, Gaasterland T, Willert K, Cappellen D. WNT5A encodes two isoforms with distinct functions in cancers. PLoS One. 2013;8(11):e80526.

74. Fisher SA. Vascular smooth muscle phenotypic diversity and function. Physiol Genomics. 2010;42A(3):169-87.

75. Helper DJ, Lash JA, Hathaway DR. Distribution of isoelectric variants of the 17,000-dalton myosin light chain in mammalian smooth muscle. J Biol Chem. 1988;263(30):15748-53.

76. Chandrashekar DS, Bashel B, Balasubramanya SAH, Creighton CJ, Ponce-Rodriguez I, Chakravarthi B, et al. UALCAN: A Portal for Facilitating Tumor Subgroup Gene Expression and Survival Analyses. Neoplasia. 2017;19(8):649-58.

77. Chandrashekar DS, Karthikeyan SK, Korla PK, Patel H, Shovon AR, Athar M, et al. UALCAN: An update to the integrated cancer data analysis platform. Neoplasia. 2022;25:18-27.

**Supplementary Figure Legends**

**
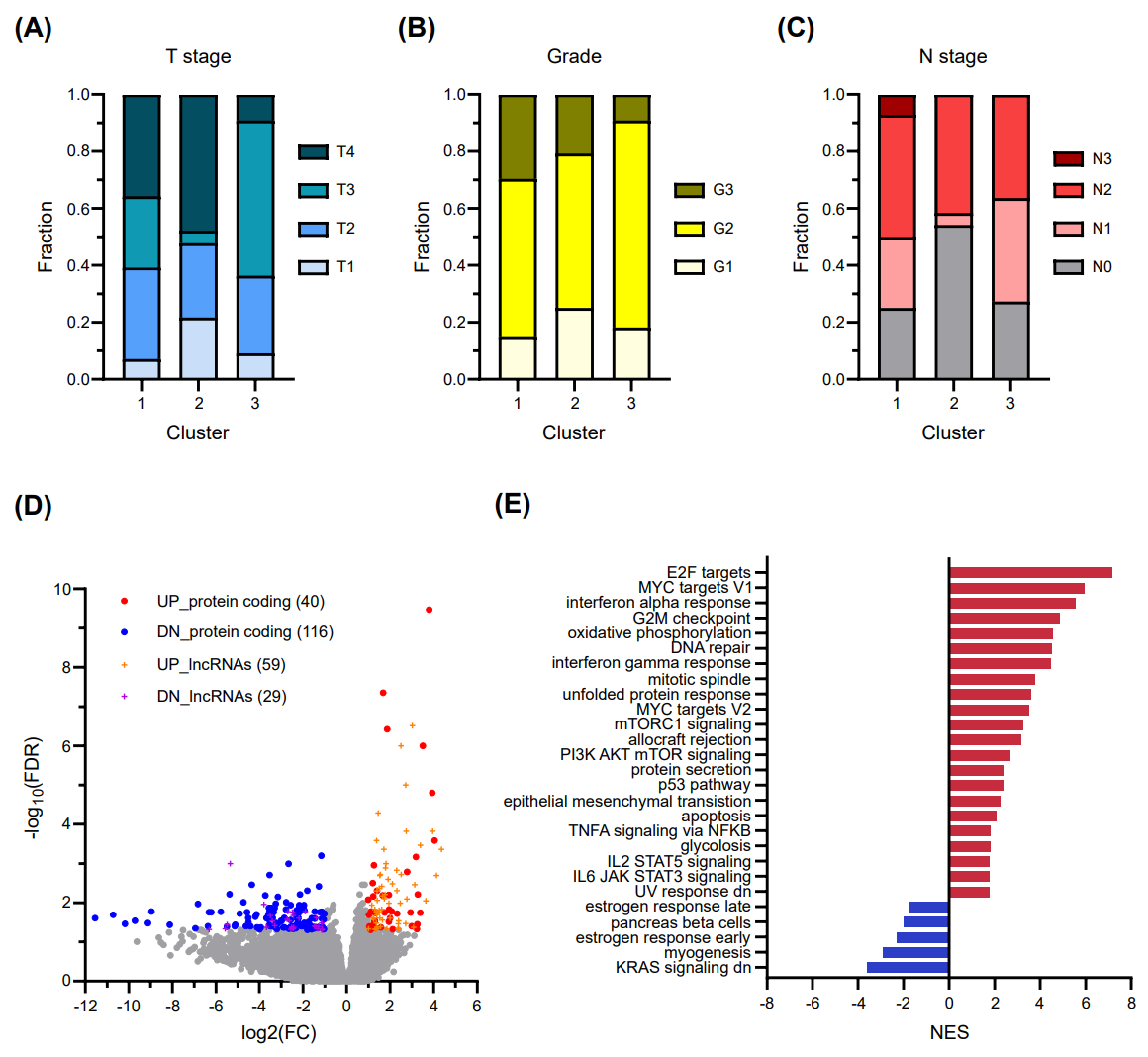
**

**Supplementary Figure S1.** **Transcriptome-based clustering is not correlated with clinical parameters.**

**(A-C)** Distribution of patients with tumors unambiguously assigned to one cluster and their respective stage (**A**), grade (**B**), and nodal status (**C**).

**(D)** Volcano plot showing genes differentially expressed between cluster 3 and combined clusters 1/2. Upregulated and downregulated genes with FDR ≤ 0.05 and |log2(FC)| ≥ 1 are highlighted.

**(E)** Overview of significantly enriched gene sets in cluster 3 (FDR ≤ 0.05) based on gene set enrichment analysis.

Abbreviations: DN, downregulated; FC, fold change; NES, normalized enrichment score; UP, upregulated.

**
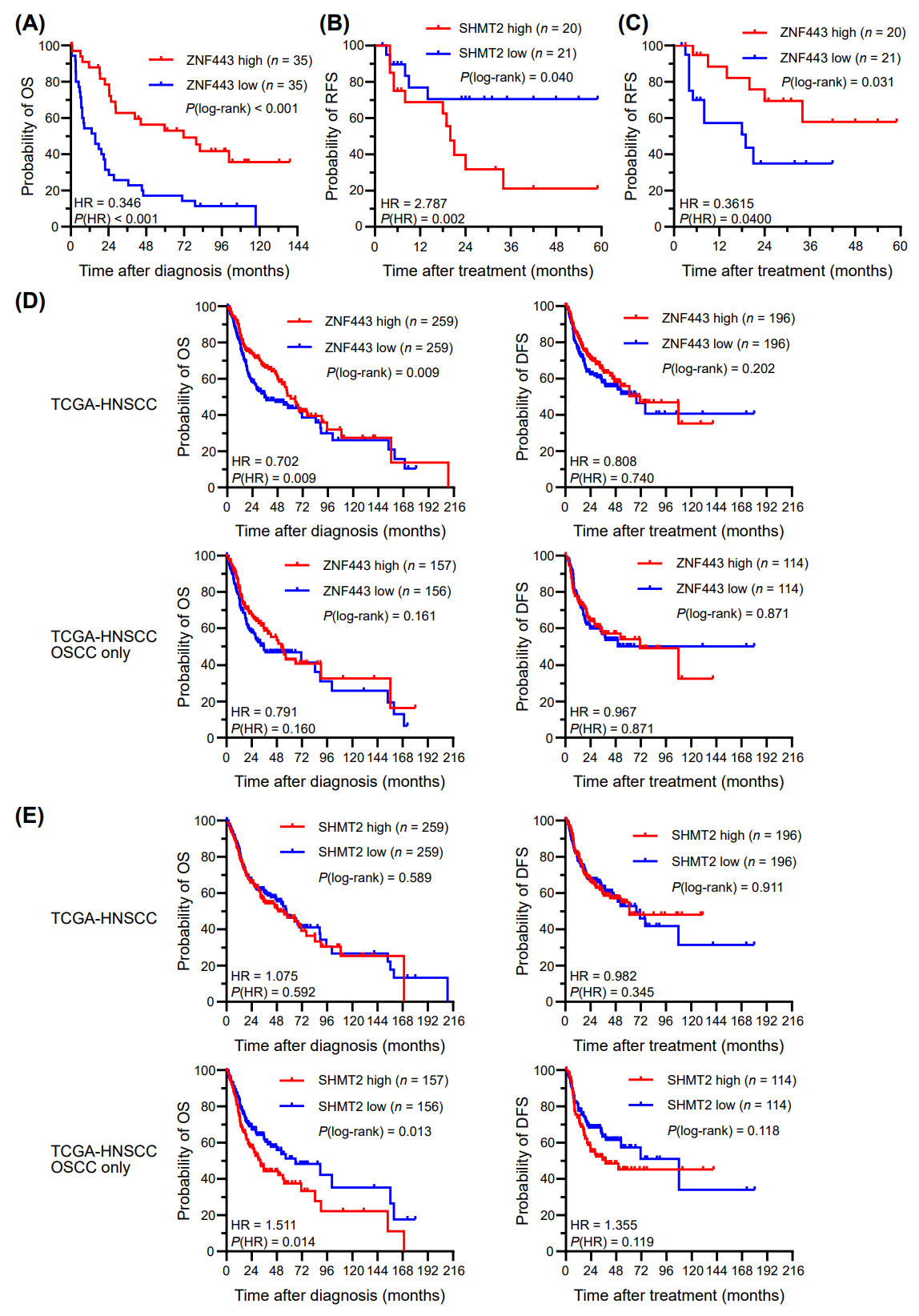
**

**Supplementary Figure S2.** ***SHMT2* has prognostic relevance only in the OSCC subgroup of the TCGA cohort.**

**(A)** Kaplan-Meier analysis of overall survival (OS) in OSCC cohort (*n* = 70 patients) stratified by *ZNF443* mRNA abundance. Samples were divided by median separation.

**(B-C)** Kaplan-Meier analysis of recurrence-free survival (RFS) in the OSCC cohort (*n* = 41 patients) using median-based stratification by *SHMT2* (**B**) and *ZNF443* (**C**) expression.

**(D-E)** Association of *ZNF443* (**D**) and *SHMT2* (**E**) expression with OS (*n* = 518 samples) and disease-free survival (DFS, *n* = 392 samples) in the TCGA-HNSCC cohort was analysed. Samples derived from anatomical subsites (alveolar ridge, buccal mucosa, floor of mouth, hard palate, lip, oral cavity, and tongue) were selected to focus specifically on OSCC samples (OS: *n* = 313, DFS: *n* = 228). For each analysis, samples were split according to the median RNA expression.

Abbreviations: DSF, disease-free survival; HNSCC, head and neck squamous cell carcinoma; HR, Hazard ratio; OS, overall survival; OSCC, oral squamous cell carcinoma; RFS, recurrence-free survival; SHMT2, Serine Hydroxymethyltransferase 2; TCGA, The Cancer Genome Atlas; ZNF443, Zinc Finger Protein 443.

**
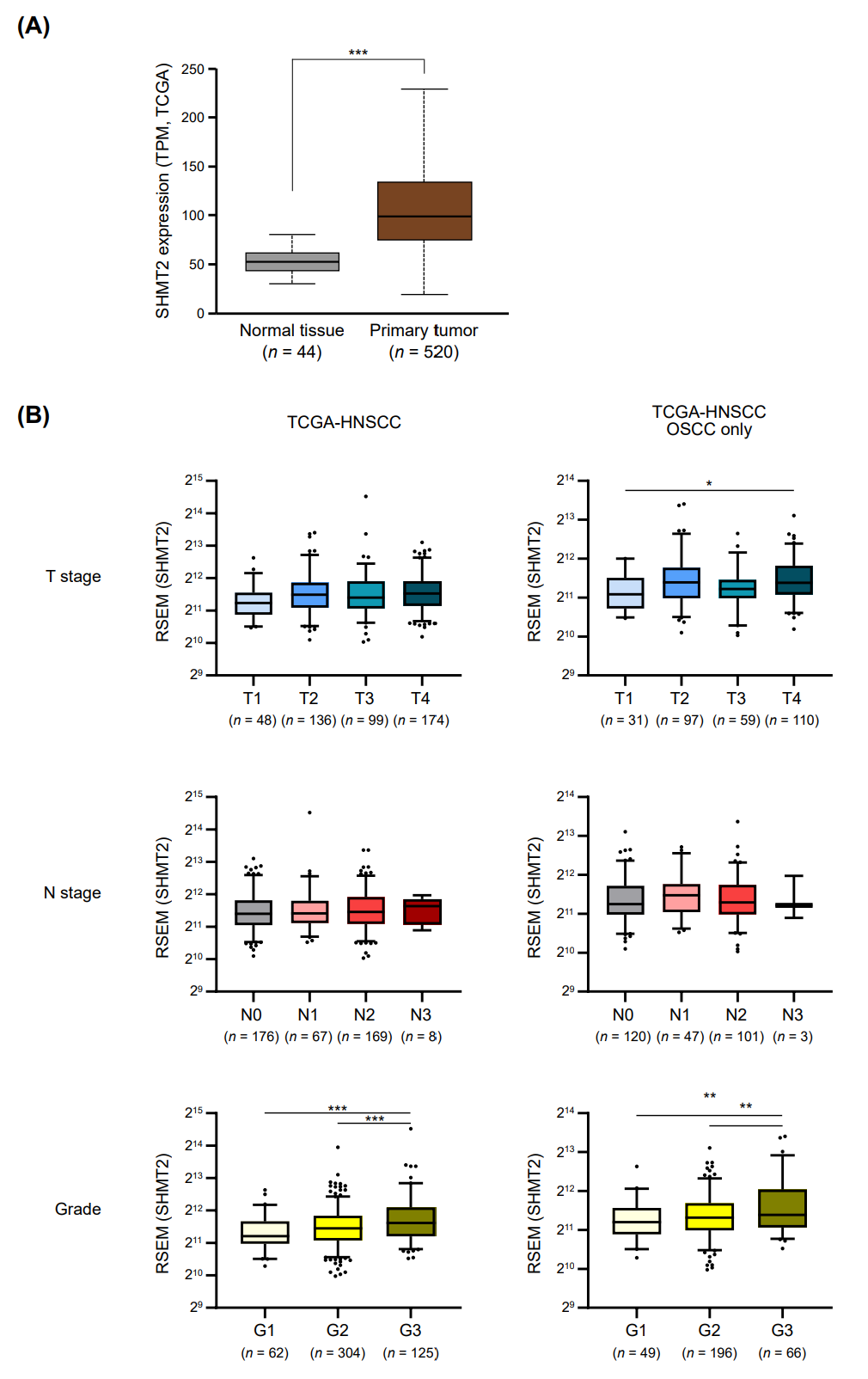
**

**Supplementary Figure S3.** **Expression of SHMT2 in TCGA-HNSCC and TCGA-OSCC cohort.**

(**A**) *SHMT2* mRNA expression in normal and tumor tissues. Whiskers show the minimum and maximum. Data were obtained from the UALCAN web resource (https://ualcan.path.uab.edu/) [76, 77]. *SHMT2* expression is higher in tumors compared to normal tissues.

**(B)** *SHMT2* transcript abundance was analysed in tumors from the TCGA-HNSCC cohort. Expression in all tumors or OSCC-only tumors of different T stages, N stages, and grades was plotted. Whiskers indicate the 5% and 95%. One-way-ANOVA (Holm-Sidak corrected) was performed for comparisons between groups (**P* ≤ 0.05, ***P* ≤ 0.01, ****P* ≤ 0.001). In the OSCC subtype, *SHMT2* expression is significantly higher in T4 tumors compared to T1 tumors. A general trend towards higher *SHMT2* expression is also observed with increasing tumor grades.

Abbreviations: HNSCC, head and neck squamous cell carcinoma; OSCC, oral squamous cell carcinoma; RSEM, RNA-seq by Expectation-Maximization; SHMT2, Serine Hydroxymethyltransferase 2; TPM; transcript per million.


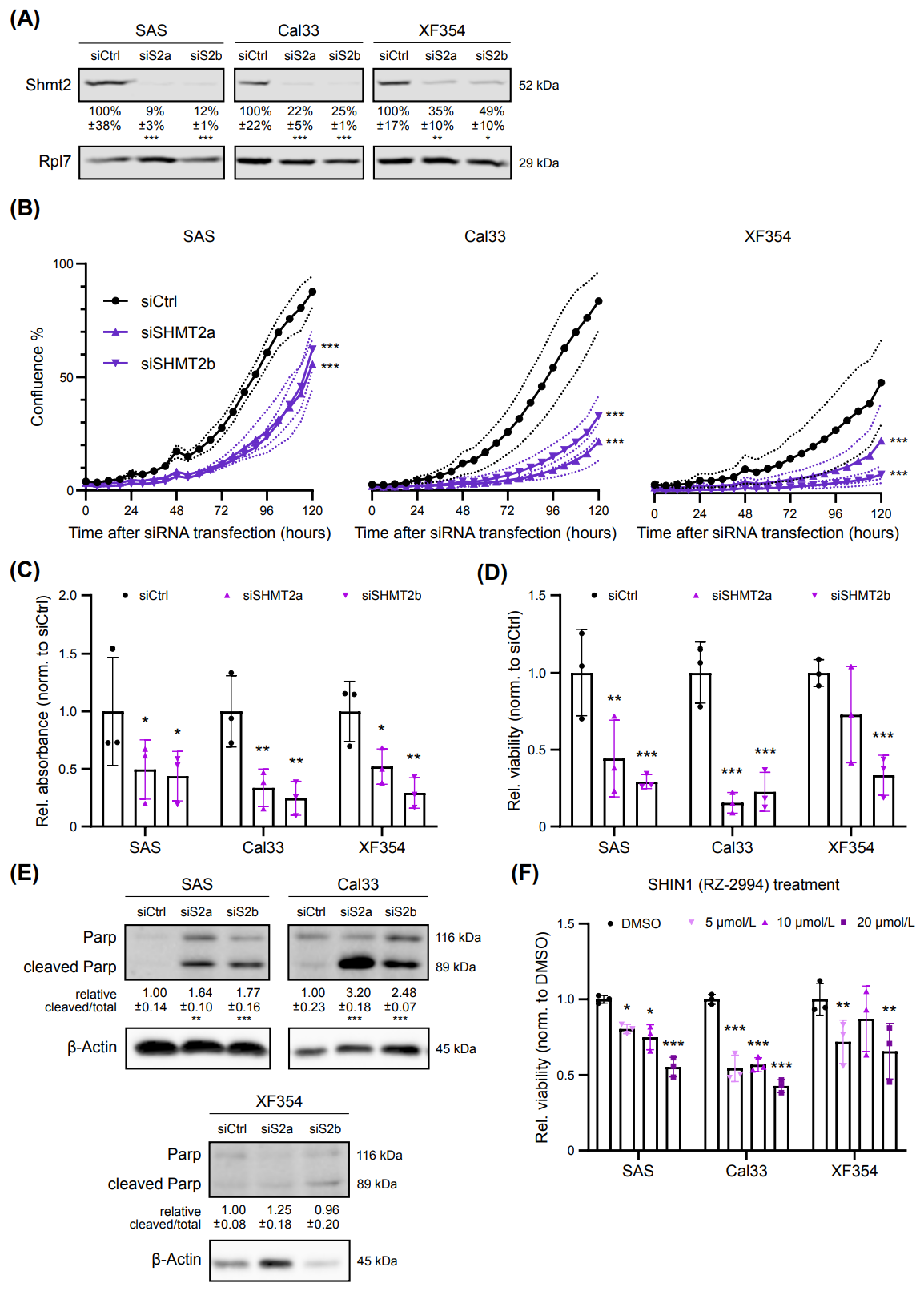


**Supplementary Figure S4.** ***SHMT2* downregulation affects proliferation, viability, and apoptosis of oral cancer cells.**

**(A)** Western blot confirms efficient siRNA-mediated depletion of SHMT2 protein in SAS, Cal33, and XF354 cells. RPL7 served as the loading control. Representative Western blot is shown (*n* = 3).

**(B)** Proliferation of SAS, Cal33, and XF354 cells was analyzed using an IncuCyte® S3 Live Cell Analysis System to calculate confluences. Mean and standard deviation (dashed line) are shown.

**(C)** BrdU assay to measure cell proliferation/cell cycle progression 72 hours after siRNA transfection.

**(D)** Cell viability was measured via MTT assay 96 hours post-knockdown.

**(E)** Representative Western blot analysis (*n* = 3) showing total and cleaved PARP to evaluate apoptosis 48 hours after siRNA transfection.

**(F)** Viability assay (MTT) of cell lines treated with indicated concentrations of the SHMT2 inhibitor (SHIN1) for 96 hours. Bar graphs show mean and standard deviation. Two-way-ANOVA (Holm-Sidak corrected) was performed for comparisons with siCtrl and DMSO control (*n* = 3; **P* ≤ 0.05, ***P*≤ 0.01, ****P* ≤ 0.001).

Abbreviations: BrdU; Bromodeoxyuridine; MTT, 3-[4,5-dimethylthiazol-2-yl]-2,5 diphenyl tetrazolium bromide; SHIN1, SHMT1/2 inhibitor 1; SHMT2, Serine Hydroxymethyltransferase 2; siRNA; small interfering RNA.


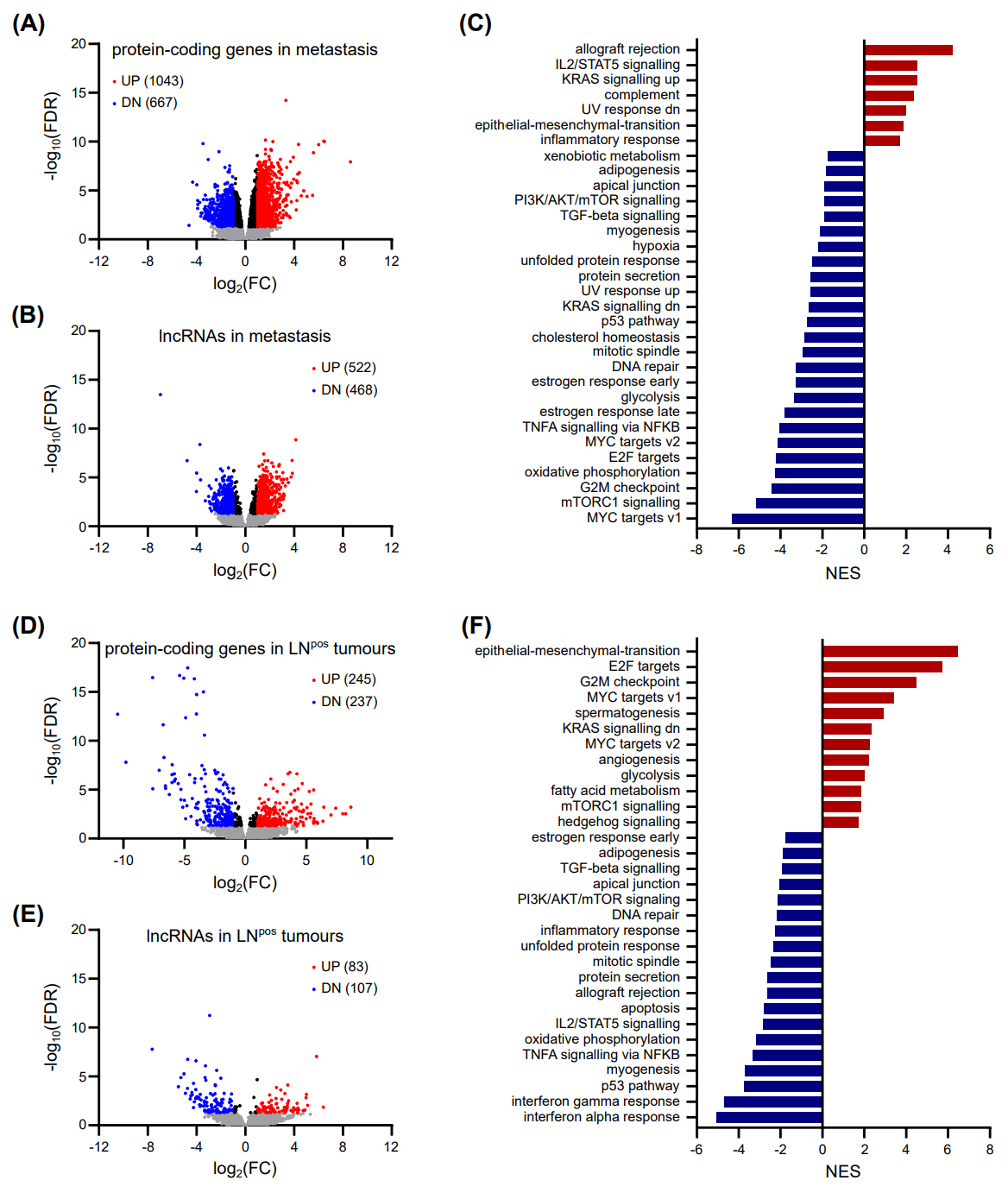


**Supplementary Figure S5.** Complementary expression analyses identify metastasis-associated genes.

**(A-B)** Volcano plots showing differentially expressed protein-coding genes (**A**) and lncRNAs (**B**) in LNM compared to their matched primary tumors (FDR ≤ 0.05; |log2(FC)| ≥ 1).

**(C)** Overview of significantly enriched gene sets in LNM according to gene set enrichment analysis.

**(D-E)** Deregulated protein-coding genes (**D**) and lncRNAs (**E**) in 43 LNM^pos^ compared to 24 LNM^neg^ primary tumors (FDR ≤ 0.05; |log2(FC)| ≥ 1).

**(F)** Significantly enriched gene sets in LNM^pos^ tumors. EMT-promoting genes showed the same positive enrichment trend in both differential gene expression analysis approaches. In contrast, IL2/STAT5 and KRAS signaling-promoting genes, as well as cell cycle-related gene sets containing E2F and MYC target genes, showed opposite enrichment in metastatic samples compared to LNM^pos^ primary tumours. In addition, some gene sets associated with processes such as angiogenesis, hypoxia, and fatty acid metabolism were specific to each analysis, potentially reflecting differences in tissue sites (lymph node vs. mucosal epithelium) as well as variations in the biology of primary tumors and metastases.

Abbreviations: DN, downregulated; EMT, epithelial-mesenchymal transition; FC, fold change; lncRNAs, long non-coding RNAs; LNM, lymph node metastasis; LNM^pos/neg^, lymph node metastasis-positive/negative; NES, normalized enrichment score; UP, upregulated.


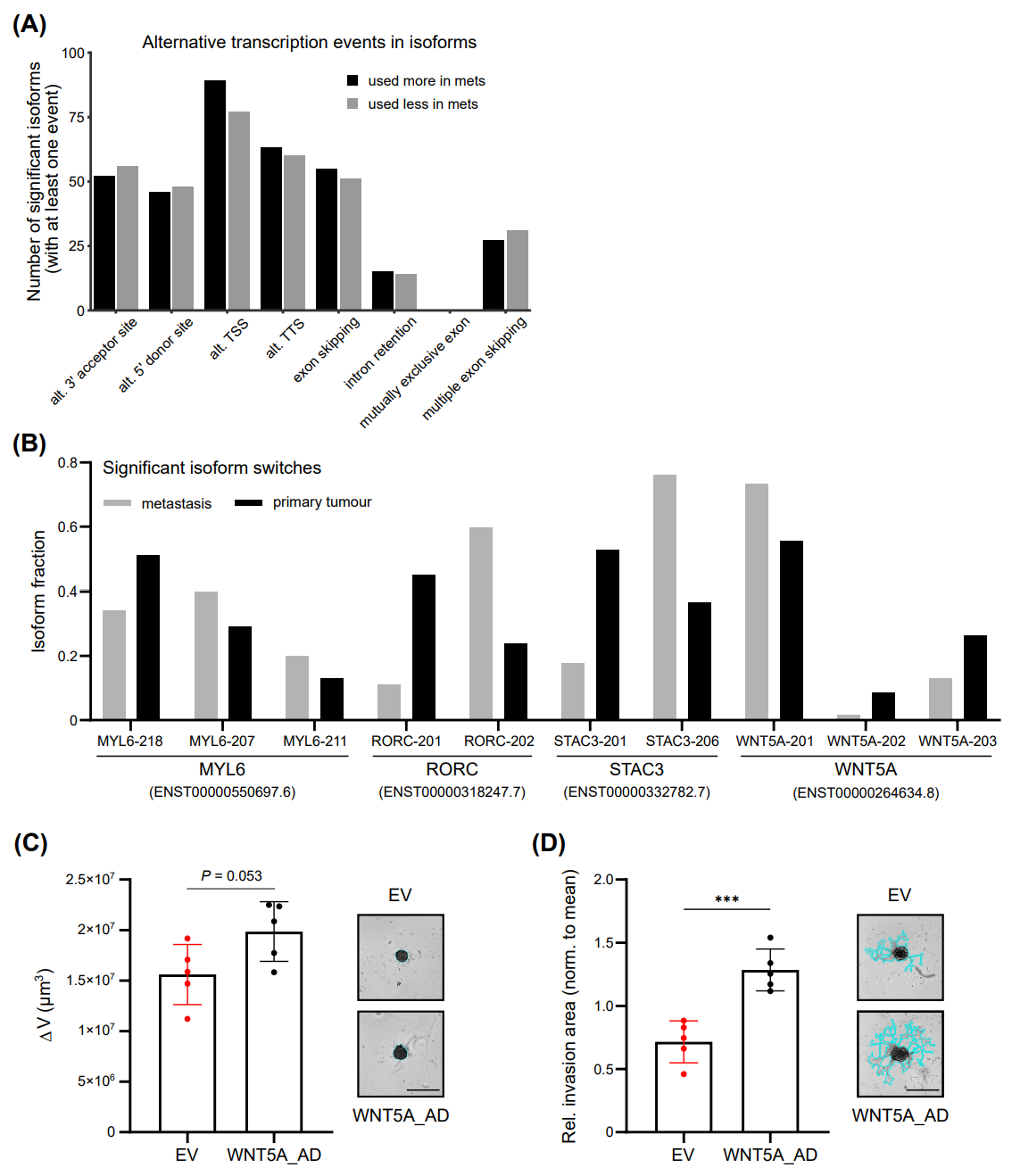


**Supplementary Figure S6. Primary tumors and metastases show isoform switches that may contribute to disease progression.**

**(A)** Overview of transcription events resulting in alternative isoform usage in LNM.

**(B)** Summary of significant isoform switches in selected genes. For example, primary tumours displayed a relatively high fraction of the canonical *MYL6* isoform (*MYL6-218*), encoding a 151 aa (~17 kDa) MYL6 protein. Exon six skipping in metastasis decreased the fraction of *MYL6-218* and increased the expression of *MYL6-207*, which encodes a 151 aa MYL6 protein differing in its C-terminal amino acid sequence. In addition, the isoform *MYL6-211*, which might encode a shortened (103 aa) MYL6 protein, also showed increased expression in LNM.

**(C-D)** Spheroid growth (**C**) and matrigel-based invasion (**D**) of SAS cells overexpressing the canonical WNTA protein (AD). Representative images are shown (48 hours after spheroid formation, scale bar represent 800 µm). Student’s t-test was used to compare two means (*n* = 5, **P* ≤ 0.05, ***P* ≤ 0.01, ****P*≤ 0.001).

Abbreviations: aa, amino acid; EV, empty vector; kDa, kilodalton; LNM, lymph node metastasis; MYL6, Myosin Light Chain 6; TSS, transcription start site; TTS, transcription termination site.

**Supplementary Tables**

**Supplementary Table S1. Differentially expressed genes in cluster 3 compared to cluster 1&2 (FDR ≤ 0.05; |log2FC |≥ 1)**

[Excel]

**Supplementary Table S2. Genes associated with overall survival in local OSCC cohort (*P* ≤ 0.05)**

[Excel]

**Supplementary Table S3. Univariate and multivariate Cox regression survival analysis in the OSCC subtype of the TCGA-HNSCC cohort.**

[Excel]

**Supplementary Table S4. Differentially expressed protein-coding genes in matched LNM compared to primary OSCC.**

[Excel]

**Supplementary Table S5. Differentially expressed non-coding genes in matched LNM compared to primary OSCC.**

[Excel]

**Supplementary Table S6. Differentially expressed protein-coding genes in LNM-positive compared to LNM-negative tumours.**

[Excel]

**Supplementary Table S7. Differentially expressed non-coding genes in LNM-positive compared to LNM-negative tumours.**

[Excel]

**Supplementary Table S8. Common deregulated genes in LNM and LNM-positive tumors.**

[Excel]

**Supplementary Table S9. Genes with significant isoform switches in matched LNM compared to primary OSCC.**

[Excel]

**Supplementary Table S10. List of primers used for cloning of *WNT5A* isoforms.**

| **Primer name** | **Direction** | **Sequence (5’ > 3’)** |
| --- | --- | --- |
| A-W5a | forward | ATGC**GCTAGC**ATGAAGAAGTCCATTGGAATATTAAGCCCA |
| B-W5a | forward | ATGC**GCTAGC**ATGGCTGGAAGTGCAATGTCT |
| C-W5a | reverse | GCAT**CTCGAG**CTACACCCTGCGGCCGGCC |
| D-W5a | reverse | GCAT**CTCGAG**CTACTTGCACACAAACTGGTCCAC |
